# Comparative Characterization of *Plasmodium falciparum* Small Heat Shock Proteins and Their Inhibition by Quercetin (3,3′,4′,5,7-Pentahydroxyflavone)

**DOI:** 10.1007/s10930-025-10281-w

**Published:** 2025-07-18

**Authors:** Francisca Magum Timothy, Tawanda Zininga

**Affiliations:** Department of Biochemistry, Stellenbosch University, Stellenbosch, 7602 South Africa

**Keywords:** Malaria, *Plasmodium falciparum*, Small heat shock proteins—sHsp, Molecular chaperones, Quercetin

## Abstract

*Plasmodium falciparum*, the parasite responsible for the most severe form of malaria, encodes three small heat shock proteins (sHsps), PfHsp20a, PfHsp20b, and PfHsp20c, each containing a conserved α-crystallin domain (ACD). These class I sHsps are hypothesized to play critical roles in proteostasis under stress, yet their specific functions have remained poorly defined. In this study, all three sHsps were recombinantly expressed and purified for structural and functional characterization. Circular dichroism and thermal shift assays revealed distinct conformational properties, with PfHsp20a exhibiting the highest thermal and chemical stability. Functional assays using malate dehydrogenase and citrate synthase confirmed that all three isoforms possess autonomous chaperone activity, although with varying efficiency. Notably, the plant-derived flavonoid quercetin disrupted both the structure and function of the sHsps in a concentration-dependent manner, with PfHsp20c being the most sensitive. Quercetin also inhibited the growth of *P. falciparum* Nf54 and Dd2 strains in vitro with IC_50_ values of 5.4 μM and 7.8 μM, respectively. These results provide the first direct evidence of independent chaperone activity in *P. falciparum* sHsps and highlight their vulnerability to small molecule inhibition. This establishes their potential as novel drug targets for antimalarial intervention.

**Supplementary Information:**

The online version contains supplementary material available at 10.1007/s10930-025-10281-w.

## Introduction

Malaria caused by *Plasmodium falciparum* parasites is still a significant global health challenge [1]. The parasite leads a complicated life in two distinct hosts, a homeothermic human and poikilothermic mosquito. It encounters physiological stress in these different environments and for survival and propagation, the parasite relies on a robust protein quality control system to prevent protein misfolding and proteotoxicity [2]. Heat shock proteins (Hsp) are molecular chaperones that play a central role in cell protein quality control by maintaining protein folding and preventing protein aggregation [3]. This coordinated action of Hsps ensures that parasite proteins maintain their functionality in the physiologically challenging environments.

Small Hsps (sHsps) are among the smallest members in molecular mass (20–40 kDa) which are ubiquitous across prokaryotes and eukaryotes [4]. Although, there are Hsp40s of ~ 40 kDa in size, they are not regarded as sHsps. Hsp40s, also known as J-domain containing proteins (JDPs), are mainly distinguished from sHsp based on the presence of a conserved J domain, which is absent in sHsps [5]. In other organisms, sHsps have been shown to play important roles in cellular protein quality control systems [6]. The sHsps are considered the initial defence mechanism against conditions that threaten the stability of the cellular proteome [7]. These sHsps are characterized by several distinct properties that differentiate them from other Hsp families. Notably, small Hsps operate in an ATP-independent holdase manner as they lack folding capability, which contrasts with other chaperones that require ATP for folding function [6, 8]. This unique feature makes sHsps especially valuable in cellular environments subjected to extreme stress, such as heat shock or oxidative damage with depleted ATP supply [9]. Another defining characteristic of sHsps is their ability to dynamically assemble and reassemble into oligomeric complexes [10]. The building blocks of these large oligomeric complexes are the homo- or heterodimers [11]. This dynamic oligomerization is critical for their function, as it allows sHsps to rapidly adapt to changing stress conditions by altering their structural configurations. Existing evidence suggests that smaller oligomers function as more active chaperones [4]. Therefore, a shift in the equilibrium of the sHsps ensemble allows for the regulation of their chaperone activity [12, 13].

Most sHsps are characterized by a conserved α-crystallin domain (ACD) [14–17]. The ACD consist of 80–100 residues and forms a compact β-sandwich structure with 7–8 antiparallel β sheets [14]. On both sides of the ACD are the variable and intrinsically disordered N-terminal and C-terminal domains (NTD and CTD), which contribute to the functional diversity of sHsps [6]. The NTD is the most divergent region among sHsps, both in length and sequence and it is intrinsically disordered [4]. The NTD contains little secondary structure, although short α-helices and β-structural elements have been identified in some members [11]. The CTD, typically under 20 residues, usually contain a conserved IXI/-V motif essential for oligomerization [18, 19]. Despite variability in sequence and oligomeric compositions of sHsps, the conserved ACD plays a central role in maintaining their structural organization [8]. Substrate recognition by sHsps from different organisms suggest a preference for translation-related proteins and metabolic enzymes [6, 13]. The interaction between sHsps and substrates appears to depend on charge and hydrophobicity, with multiple binding sites throughout the protein known as multivalent interactions which are present in dynamic intrinsically disordered proteins also contributing [13]. The NTD and ACD of sHsps are implicated in substrate binding, featuring solvent-exposed hydrophobic patches available for interaction [20]. It should be noted that sHsp in eukaryotes lack refolding capability and need the larger families of Hsp70/Hsp90 chaperone complex for substrate refolding [10].

In the search for new antimalarial agents, attention has turned to plant-derived compounds such as quercetin (3,3′,4′,5,7-pentahydroxyflavone). Quercetin is a flavonoid found in various fruits and vegetables and is well known for its broad biological activities, including antioxidant, anti-inflammatory, and anticancer properties [21]. Importantly, quercetin has demonstrated anti-plasmodial efficacy [22], showing potential against *P. falciparum* and other protozoan parasites such as *Trypanosoma brucei*. In *T. brucei*, quercetin inhibits the ATPase activity of Hsp70 [23], highlighting its ability to target essential molecular chaperones involved in parasite survival. By targeting these molecular chaperones, quercetin could disrupt the proteostasis network within *P. falciparum*, thereby impairing its survival and pathogenicity. Given the growing challenge of antimalarial drug resistance, the exploration of compounds like quercetin could pave the way for new antimalarial strategies.

The * P. falciparum* genome encodes three distinct isoforms of sHsps, annotated as PF3D7_1304500 (PfHsp20a), PF3D7_0816500 (PfHsp20b), and PF3D7_1211100 (PfHsp20c) [19]. Despite their predicted roles in proteostasis and cellular stress response, these class I sHsps remain poorly characterized, particularly in contrast to the extensively studied large Hsp families in the parasite. In this study, we successfully expressed and purified full-length recombinant forms of all three *P. falciparum* Hsp20s, enabling the first comprehensive biochemical and functional characterization of these proteins. Additionally, we investigated the impact of quercetin, as a model small-molecule inhibitor targeting the structural and functional integrity of *P. falciparum* Hsp20 proteins. This approach provides a foundational framework for the rational design of novel antimalarial therapies aimed at disrupting parasite proteostasis by targeting the sHsp machinery.

## Materials and Methods

### Materials

Unless otherwise specified the chemical reagents used in this study were procured from Thermo-Fisher Scientific (MA, USA), Merck (Darmstadt Germany), Sigma-Aldrich, (CA, USA). The HRP conjugated His-Probe used to validate the expression of the proteins was purchased from Thermo Scientific (IL, USA).

### Plasmid Construct Designing

The codon harmonised plasmid constructs coding for the expression of PfHsp20a with the PlasmoDB and Uniport accession number (PF3D7_1304500; Q8IES0, residues 1–211), PfHsp20b (PF3D7_0816500; Q8IB02, residues 1–173), and PfHsp20c (PF3D7_1211100; Q8I5T9, residues 1–231) were synthesised by GenScript (NJ, USA). The synthesized target genes were inserted between *Bam*HI and *Hind*III restriction sites. These genes were cloned into the pQE30 vector, resulting in pQE30/PfHsp20a, pQE30/PfHsp20b, and pQE30/PfHsp20c constructs with N-terminal 6xhis tag, respectively. The plasmid constructs were codon optimised for heterologous expression of PfHsp20a, PfHsp20b and PfHsp20c proteins in *E. coli* expression systems. The integrity of the plasmid constructs was confirmed using restriction enzyme digestion and validated by gene sequencing. The signal peptides predictions were conducted using deeploc2.1 (https://services.healthtech.dtu.dk/).

### Overexpression of Recombinant PfHsp20a, PfHsp20b and PfHsp20c

Chemically competent *E. coli* XL1 Blue cells were separately transformed with pQE30/PfHsp20a, pQE30/PfHsp20b, and pQE30/PfHsp20c. Overexpression of the recombinant proteins were induced with 0.5 mM isopropyl β-D-1-thiogalactopyranoside (IPTG) for 16-h at 18 °C. The cells were harvested by centrifugation at 8000 × *g* for 20 min at 4 °C (Avanti J-E centrifuge, Beckman Coulter, USA). The cell pellets were lysed using lysis buffer (20 mM Tris–HCl, pH 7.4, 500 mM NaCl, 5 mM imidazole), followed by sonication (W-385 Sonicator Ultrasonic Processor + C3 20 kHz Converter probe, Heat Systems Ultrasonics, Inc., USA) using six cycles of 1-min pulses with 1-min pauses at 50% amplitude. The soluble and insoluble fractions were separated by centrifugation at 20,000 × *g* for 30 min at 4 °C. The supernatant was clarified by filtration through a 0.45 µm GVS acrylic single-use syringe filter (Lasec, South Africa) and loaded onto an Immobilized Metal Affinity Chromatography (IMAC) connected to an AKTA Go™ fast protein liquid chromatography system (FPLC) (Cytiva, MA, USA) as previously described [24]. The bound proteins were eluted using elution buffer (20 mM Tris–HCl, pH 7.4, 500 mM imidazole, 500 mM NaCl). The purified proteins were extensively dialysed by spinning in an Amicon® Ultra 10 kDa MWCO Centrifugal filter column at 4,000 × *g* for 1 h at 4 °C in TBS buffer (20 mM Tris–HCl, pH 7.4, 300 mM NaCl supplemented with 50% (v/v) glycerol and 1 mM PMSF). The protein samples were flash frozen in liquid nitrogen and stored at -80 °C until use. The expression and purification samples were analysed with SDS-PAGE and validated via Western blot analysis using a HRP conjugated His-Probe™ (ThermoScientific, IL, USA). The approximate purity of each recombinant protein was estimated by densitometric analysis of SDS-PAGE gels using the GelAnalyzer tool in ImageJ (https://imagej.net/downloads).

### Investigation of *P. falciparum* Hsp20s Self-association

To determine the capability of the parasite sHsps to self-associate, dynamic light scattering (DLS) was conducted as previously described [25] with modifications. The DLS measurements were performed using the Zetasizer Nano S® (Malvern Instruments; Worcestershire United Kingdom), which utilizes a backscatter detection system at 173°, close to the incident light angle of 180°. For each measurement, varying concentrations of 0–6 µM of PfHsp20a / PfHsp20b/ PfHsp20c suspended in DLS buffer (20 mM Tris, pH 7.4, 300 mM NaCl) were each separately added to a clean quartz cuvette and allowed to equilibrate at 25 °C for 10 min before measuring the hydrodynamic radius (Rh) [26]. To determine the effect of inhibitors on the oligomerization of *P. falciparum* Hsp20s, the assay was repeated in the presence of 3 µM quercetin. The refractive index of the DLS buffer was determined using an Abbe refractometer and was found to be 1.34 to give a viscosity at 25 °C of 0.879 mPa·s which was used for data processing calculations using Eq. 1. Since light scattering depends on both the refractive index of macromolecules and the solvent viscosity, these properties were incorporated into the calculation of the z-averaged corresponding to Rh, by the Zetasizer Nano S software (Malvern Instruments; Worcestershire United Kingdom) and the calculation utilizes the Stock-Einstein equation:

1 $$ {\text{Rh}} = {\text{k}}_{{\text{B}}} {\text{T}}/{6}\pi \eta {\text{D}} $$

where Rh represent the hydrodynamic radius, k_B_ representing the Boltzmann constant, T represent absolute temperature in kelvin, η representing the viscosity and D representing the diffusion coefficient. All the Rh distribution measurements were performed in triplicates repeated three times and BSA was used as a control with known Rh values. The approximate oligomer sizes were calculated using the average predicted Rh values from DLS and the fluidic webserver (https://fluidic.com/hydrodynamic-radius-calculations/) using the respective UniProt accession numbers.

### Secondary Structure Analysis Using Circular Dichroism (CD) Spectroscopy

The secondary structure of the recombinant *P. falciparum* Hsp20s was investigated using circular dichroism (CD) spectroscopy as previously described [27]. The CD spectra were recorded between 190 and 240 nm using a ChiraScan™ plus CD instrument (Applied Photophysics, MA, USA). Briefly, 2.0 μM of each protein suspended in CD buffer (10 mM KH_2_PO_4_, 100 mM KF, pH 7.5) was analysed in a quartz cuvette (Hellma Analytics, Germany, Müllheim) with a pathlength of 1.0 mm. The CD spectra were averaged over 15 scans after baseline correction before being smoothened.

To investigate the capability of the *P. falciparum* Hsp20s to withstand heat stress, the CD measurements were repeated at varying temperatures as previously described [24]. Briefly, the thermal stability of the recombinant proteins was assessed by ramping up the temperature from 25 °C to 90 °C at a rate of 1 °C/min of the protein suspended in CD buffer whilst taking readings. We monitored the capability of the recombinant proteins to refold to their original structure after thermal stress exposure by cooling the temperature from 90 °C to 25 °C. In addition, we assessed the chemical stress resilience of the recombinant proteins. The recombinant proteins were suspended in buffer with varying concentration 0–8 M of urea or 0–6 M guanidine hydrochloride (Gdn-HCl) for 1 h prior to CD measurements. In each case, the generated CD spectra were averaged after baseline correction (CD reading of buffer with constituents [urea/Gdn-HCl] without protein). The effect of inhibitors on the structure of *P. falciparum* Hsp20s was investigated by repeating the assay in the presence of 3 μM quercetin. Data analysis to compare the relative structural changes for thermal unfolding/refolding and chemical unfolding, the molar residue ellipticity (θ) obtained at 195 nm and 205 nm was converted to the fraction of folded proteins at any given temperature as a ratio of signal at 25 °C using Eq. 2 as previously described [24].

2 $$ {\text{Folded}}\;{\text{fraction}} = \left( {\theta_{s} - \theta_{90} } \right)/\left( {\theta_{25} - \theta_{90} } \right) $$

where θs is the mean residue ellipticity (MRE) of the protein sample at any given temperature/(urea/Gdn-HCl) concentration, θ_90_ is the MRE at 90 °C or highest (urea/Gdn-HCl) concentration and θ_25_ is the MRE at 25 °C or lowest (urea/Gdn-HCl) concentration.

### Investigation of Thermal Stability of the Recombinant Proteins Using Differential Scanning Fluorimetry 

To investigate the thermal stability of *P. falciparum* Hsp20s structure, differential scanning fluorimetry (DSF) was conducted as described [28]. The assay was initiated by mixing 10 µM of recombinant protein in TBS buffer supplemented with 20 × SYPRO Orange protein dye (Merck, Johannesburg, SA) in PCR strip tubes and loaded onto a Mic qPCR magnetic induction cycler (Thermo-Fisher Scientific, USA). The thermal denaturation assay was conducted at a temperature range of 40–95 °C, at an increment of 0.2 °C/s. The assay was conducted in the presence of 3 μM quercetin to determine the effect of inhibitor on the thermal resilience of the proteins. Data acquisition was performed using the Mic qPCR 2.12.6 software (Thermo-Fisher Scientific, USA), and the results were analysed following the previously described method [29]. The raw fluorescence data from the melting curves were analysed after double referencing of baseline, signal of dye in buffer and of the background signal for each experiment (the minimum value from all points in each plotted curve). The first derivative of the normalized fluorescence was also calculated using Eq. 3, to determine the peak thermal shift (inflection point) using Eq. 4 and the results were plotted using GraphPad Prism v10.3.1 (GraphPad Software, CA, USA) as previously described [28]*.*

3 $$ Y = \frac{{Bottom + \left( {Top - Bottom} \right)}}{{1 + e^{{\left( {\frac{Tm - x}{{slope}}} \right)}} }} $$

4 $$ \begin{aligned} \frac{{dF}}{{dT}} = & \frac{{Bottom + \left( {Top - Bottom} \right)}}{{1 + ~e^{{\left( {\frac{{Tm - x}}{{slope}}} \right)}} }} \\ & \quad \times ~\left( {1~ - \frac{{Bottom + \left( {Top - Bottom} \right)}}{{1 + ~e^{{\left( {\frac{{Tm - x}}{{slope}}} \right)}} }}} \right) \\ \end{aligned} $$

“Bottom” is the lowest value of the melting curve, “Top” is the peak melting curve value, and Tm is the inflection point (°C).

### Investigation of the Chaperone Function of *P. falciparum* Hsp20s Using Malate Dehydrogenase and Citrate Synthase Assay

The capability of the recombinant *P. falciparum* Hsp20s to suppress the thermal induced aggregation of Malate dehydrogenase (MDH) from pig heart (Roche Diagnostics, Germany) was conducted as previously described [30]. The assay was initiated by mixing 2 µM MDH and varying concentrations of the purified recombinant *P. falciparum* Hsp20s in TBS buffer (20 mM Tris–HCl, 300 mM NaCl, pH 7.4). The mixed reactions were incubated at 45 °C for 2 h whilst simultaneously measuring the absorbance at 360 nm every 10 min to monitor changes in light scattering using a Multiskan Sky Microplate Spectrophotometer (Thermo-Fisher Scientific, USA). As a control, 5.0 µM BSA was mixed with 2.0 µM MDH. To further validate the chaperone function of the parasite sHsps, the experiment was repeated using citrate synthase (CS) from porcine heart as model substrate using a previously described protocol [31]. To determine *P. falciparum* Hsp20 sensitivity to inhibitor, the chaperone activity assay was repeated in the presence of increasing concentration of 0.75 µM–3 µM quercetin. The absorbance changes of uninhibited MDH/CS aggregation without any chaperones were used for normalising the data which was set at 100% aggregation. The data was analysed after baseline subtraction (absorbance of buffer without chaperones). All experiments were performed in triplicate and repeated three times.

### Investigation of Liquid–Liquid Phase Separation of *P. falciparum* Hsp20s

The propensity of the parasite Hsp20s to form liquid–liquid phase separation was predicted using the FuzDrop webtool available at https://fuzdrop.bio.unipd.it/predictor [32]. Briefly, the amino acid sequences for PfHsp20a, PfHsp20b and PfHsp20c were retrieved from PlasmoDB and uploaded in fasta format to analyse the propensity of the proteins to form liquid–liquid phase separation protein condensates on FuzDrop webtool [33].

### Molecular Docking

After observing the potential inhibitory effect of quercetin on the *P. falciparum* Hsp20s biochemical activities, we sought to evaluate the potential direct binding poses of quercetin using molecular docking as previously described [27]. The protein/receptor were processed using the Schrödinger Receptor Grid Generation tool. Glide docking was used to study the binding affinity and nature of the interaction between the ligand and receptor. Molecular docking was performed with the Schrödinger docking program, Glide, and multiple conformers of the ligand were generated during the docking process. To optimize computational efficiency, receptors were treated as rigid structures, while ligands were allowed flexibility to mimic in vivo conditions, and the docking complexes with the most favourable free binding energy conformations (lowest kcal/mol) were considered as the best interactions between the target receptors and ligands.

### Molecular Dynamics Simulations

Molecular dynamic (MD) simulations were performed as previously described by [27], with minor modifications. The energy-minimized docked complexes were solvated using the system builder function within the Desmond module of the Schrödinger suite 2022–1. The TIP3P solvent model was used, and a cubic box was used to specify the boundary conditions with a volume minimized 10 Å. The OPLS_2005 force field was applied, and the system was neutralized by adding Cl^−^ or Na^+^ ions according to the total charge, maintaining a salt concentration of 0.15 M. The MD simulations of the apo-proteins and *P. falciparum* Hsp20-ligand complexes were executed using Desmond. Simulations were performed under NPT ensemble conditions with a pressure of 1 bar and a temperature of 300 K for 100 ns. The prepared MD jobs were submitted to the Centre for High Performance Computing (CHPC) cluster, and each simulation was repeated three times independently.

### Antiplasmodial Activity of Quercetin

To evaluate the antiplasmodial activity of quercetin against *P. falciparum*, both the drug-sensitive (Nf54) and drug-resistant (Dd2) strains were assessed. Continuous cultures of asexual erythrocyte stage of *P. falciparum* were maintained using RPMI 1640 medium supplemented with 0.5% Albumax II, under standard conditions (37 °C, 3% O₂, 4% CO₂, balance N₂), according to the Trager and Jensen method as described in [34]. Quercetin was dissolved in 100% DMSO to create a 10 mM stock solution and serially diluted in culture medium to a final assay concentration range. The highest concentration of DMSO to which the parasites were exposed was maintained below 0.5%, a level shown to have no significant effect on parasite viability [34]. Parasite cultures were incubated in 96-well plates with test compound for 72 h under standard culture conditions and parasite viability was assessed using the parasite lactate dehydrogenase (pLDH) assay. After incubation, 15 µL from each well was transferred to a new plate containing 100 µL Malstat reagent and 25 µL nitroblue tetrazolium solution. Plates were developed in the dark for 20 min and absorbance was measured at 620 nm using a spectrophotometer. The dose response curves were generated on GraphPad prism v10.1 (GraphPad Software, USA).

### Prediction of the Pharmacological and ADME/T Properties of Quercetin

The pharmacokinetic and toxicity profiles of quercetin were predicted in silico using the Molinspiration server (v2022.08), available at https://www.molinspiration.com/cgi-bin/properties. The tool provided predictions of key ADME/T parameters, including intestinal absorption and skin permeability, based on model cell data. Distribution properties such as blood–brain barrier permeability (log BB) and central nervous system (CNS) penetration were estimated. Metabolic pathways were predicted through simulated interactions with cytochrome P450 enzymes. Excretory and toxicity liabilities were assessed by evaluating the probability of hERG channel inhibition, hepatotoxicity, and skin sensitization. In addition, the compound’s compliance with Lipinski’s Rule of Five was evaluated through calculated descriptors, including molecular weight, dipole moment, and hydrogen bond donor and acceptor counts. These predictive results provided a comprehensive profile of quercetin’s drug-like behaviour and pharmacological viability.

## Results

### Overexpression and Purification of Recombinant PfHsp20a, PfHsp20b and PfHsp20c

The recombinant PfHsp20a, PfHsp20b and PfHsp20c proteins were successfully overexpressed in XL1 Blue *E. coli* cells and purified using affinity chromatography (Supplementary Figure S1). SDS-PAGE analysis of the recombinant PfHsp20a, PfHsp20b and PfHsp20c showed species that migrated at approximately 25 kDa, 20 kDa, and 26 kDa, corresponding to their theoretical sizes, respectively, and validated by western blotting using His Probe (Supplementary Figure S1). The purity levels of the recombinant proteins were 97.6% for PfHsp20a, 87.9% for PfHsp20b, and 89.6% for PfHsp20c, based on densitometric analysis of the SDS-PAGE, thus confirming suitability for downstream analyses.

### Secondary Structure Analysis of *P. falciparum* Hsp20s

**Fig. 1:**
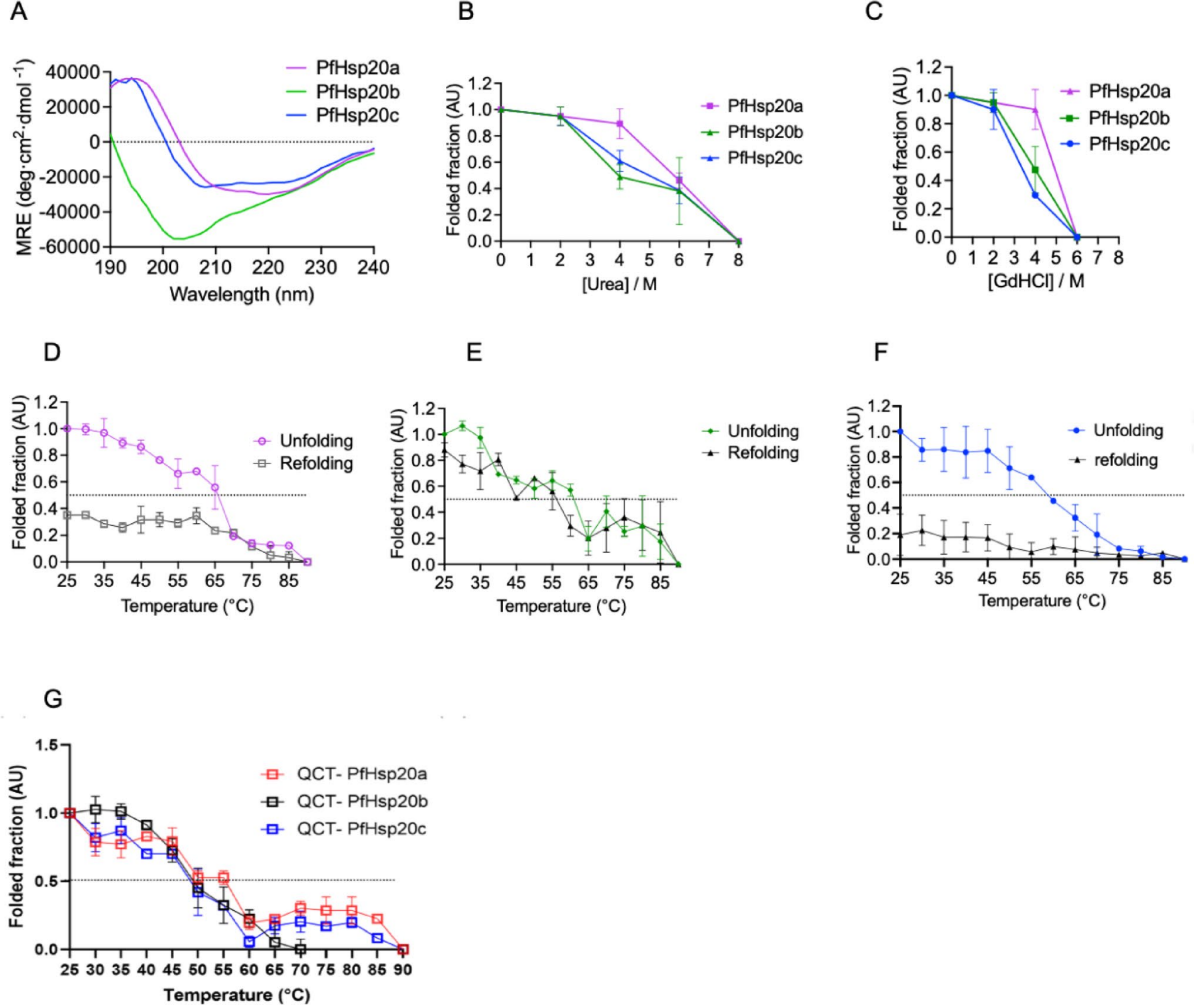
Secondary structure analysis of *P. falciparum* Hsp20s. **A** CD spectra of full length PfHsp20a, PfHsp20b and PfHsp20c. The analysis of the effect of chaotropic agents **B** urea, **C** guanidine hydrochloride. The analysis of the effect of temperature on the structure of **D** PfHsp20a, **E** PfHsp20b and **F** PfHsp20c. **G** The comparative unfolding of the *P. falciparum* in the presence of 3 μM quercetin. The folded fraction was determined as a ratio of the protein fold at any condition compared to that in native buffer with or without inhibitor at 25 °C monitored at both 195 and 205 nm. Error bars represent the SEM of the mean from three independent experiments

The secondary structure analysis of PfHsp20a, PfHsp20b and PfHsp20c was conducted using CD spectroscopy. The CD spectra for PfHsp20a and PfHsp20c registered broad minima at 218 nm consistent with a significant β-sheet structure from the ACD (Fig. 1A). PfHsp20b exhibited a unique deep minimum at 202 nm, suggesting a large number of unstructured regions presumably from the NTD and CTD composition of secondary structures, similar to AlphaFold predicted structures (Supplementary Figure S2).

In order to validate the secondary structure stability of PfHsp20a, PfHsp20b and PfHsp20c, the proteins were exposed to chaotropic agents, urea and guanidine hydrochloride (Gdn-HCl) (Fig. 1B, C). The folded fraction analysis showed a concentration dependent decrease in folded fraction for all proteins indicating that they were sensitive to urea denaturation. The three proteins were stable up to 2 M urea and started losing their fold on further increase of urea concentration. The recombinant PfHsp20a had a two-phase unfolding pattern with the first slow unfolding up to 4 M urea, which was followed by a rapid unfolding from 4 to 8 M urea. On the contrary, PfHsp20c and PfHsp20b had a more gradual unfolding from 2 to 8 M urea. Similarly, all the recombinant proteins were susceptible to Gdn-HCl induced unfolding but did not rapidly unfold up to 2 M Gdn-HCl. PfHsp20a was the most resilient of the three proteins with a two-step unfolding with an initial slow unfolding up to 4 M Gdn-HCl, followed by a more rapid unfolding after 4 M. PfHsp20b and PfHsp20c had a rapid loss of the folded structure from 2 M up to 6 M Gdn-HCl. These unique unfolding patterns suggest different structural stabilities of the three proteins when subjected to chaotropic agents.

### Thermal Unfolding and Refolding Analysis with Circular Dichroism

The impact of thermal stress on the secondary structure of *P. falciparum* Hsp20 isoforms was assessed using CD spectroscopy by monitoring ellipticity at 195 and 205 nm (Supplementary Figure S3, Fig. 1). PfHsp20a and PfHsp20c exhibited relatively high thermal stability with melting temperatures (Tm) of 65 °C and 63 °C, respectively, while PfHsp20b was less stable with a Tm of 50 °C (Table 1). Upon heat-induced unfolding and subsequent cooling from 90 °C to 25 °C, PfHsp20a regained approximately 40% of its original fold (Fig. 1D), and PfHsp20b recovered up to 85% (Fig. 1E), likely due to its disordered structure. In contrast, PfHsp20c was more susceptible to heat and recovered only 15% of its structure (Fig. 1F). These results suggest that although PfHsp20b unfolds more readily, it is also better equipped to refold after thermal stress, likely due to its flexible, unordered regions. In addition, multiple sequence alignment of the three PfHsp20 revealed low sequence identity among all three isoforms, PfHsp20a shares only ~ 23.81% identity with PfHsp20b and ~ 23.15% with PfHsp20c, while PfHsp20b and PfHsp20c share ~ 23.49% identity (Supplementary Figure S2). This low sequence similarity indicates that the three isoforms are structurally divergent from one another, which may contribute to their distinct thermal stability profiles. The substantial difference in PfHsp20a’s Tm values observed could reflect isoform-specific unfolding behaviour. This may involve early hydrophobic exposure with partial retention of secondary structure.

**Table 1:** The melting temperature of *P. falciparum* Hsp20s

Protein	CD Tm ± SD/°C		DSF Tm ± SD/°C	
	Apo	QCT	Apo	QCT
PfHsp20a	66.1 ± 3.3	55.0 ± 2.4	51.3 ± 1.7	53.9 ± 0.2
PfHsp20b	61.3 ± 6.0	50.1 ± 4.0	64.0 ± 5.1	47.1 ± 4.5
PfHsp20c	58.9 ± 1.9	50.9 ± 4.7	56.7 ± 4.5	55.9 ± 2.1

The Tm is reported as the mean value for three technical repeats. The Standard deviation (SD) of mean is shown

The effect of quercetin on the thermal stability of these proteins was also evaluated using CD (Fig. 1G). In the presence of 3 µM quercetin, significant reductions in Tm were observed: PfHsp20a and PfHsp20b decreased by 10 °C and 11 °C, respectively, while PfHsp20c dropped by 8 °C (Table 1). These findings indicate that quercetin destabilizes the secondary structure of all three PfHsp20 isoforms under heat stress, with PfHsp20b being the most affected. Overall, the data suggest that quercetin targets structural integrity in a dose and isoform specific manner, further supporting its potential as a modulator of *P. falciparum* sHsps.

### Thermal Stability Using Differential Scanning Fluorimetry

**Fig. 2:**
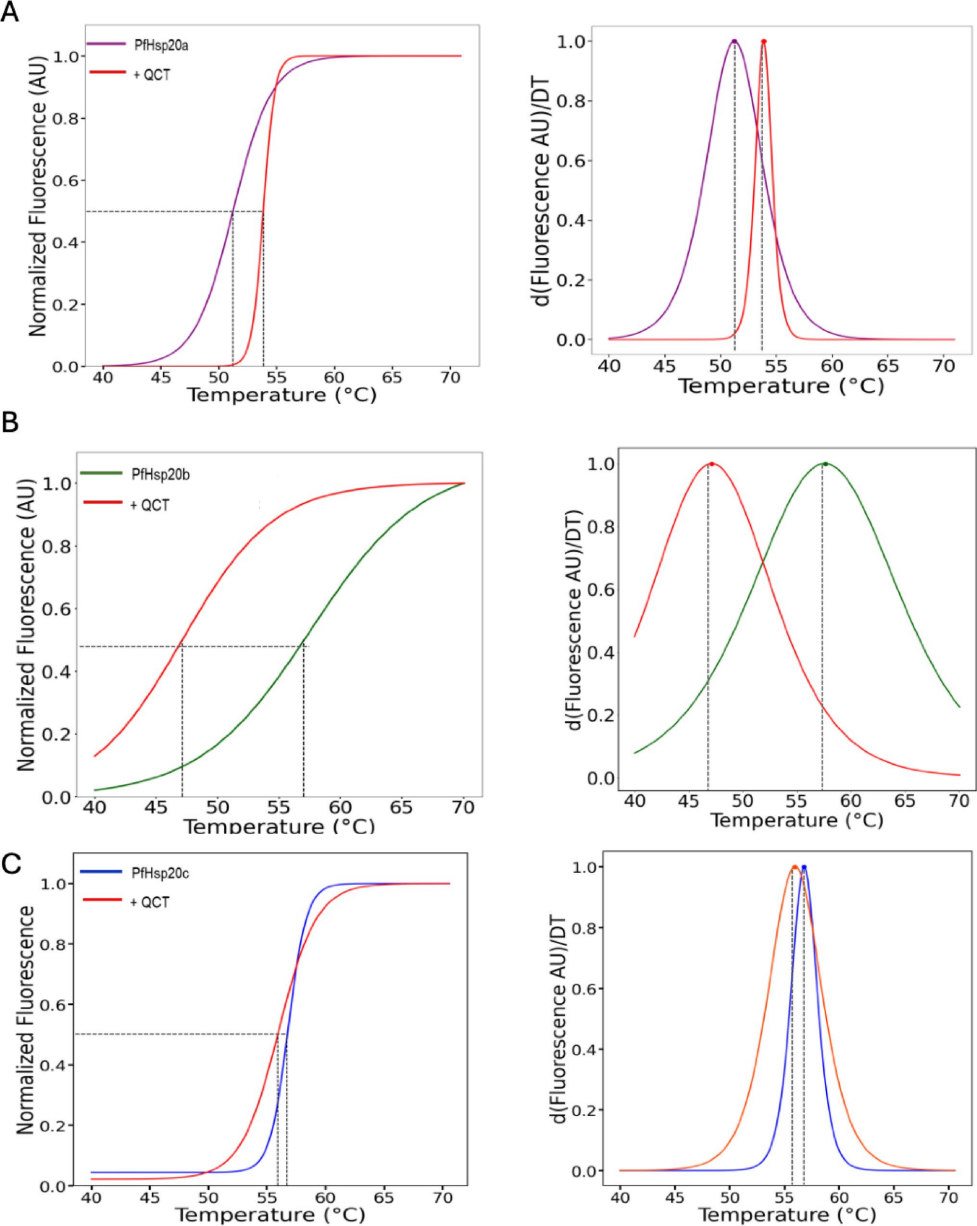
Thermal shift analysis of PfHsp20a, PfHsp20b and PfHsp20c. Sigmoidal curves for PfHsp20a (**A**), PfHsp20b (**B**), PfHsp20c (**C**). *P. falciparum* Hsp20s. The inflexion points, which corresponds to the melting temperatures are indicated with black dotted lines. The respective first derivative plots of the Tm are shown (right panel). The red sigmoid curves/peaks indicate presence of 3 µM quercetin

The structural stability of *P. falciparum* Hsp20 isoforms was assessed using DSF following secondary structure characterization. PfHsp20a, with a predominantly β-sheet composition, showed a melting temperature (Tm) of 51.3 °C, followed by PfHsp20c with a Tm of 56.7 °C (Table 1). Surprisingly, PfHsp20b, despite being predominantly unordered, exhibited the highest thermal stability with a Tm of 64.0 °C (Fig. 2A, Table 1). These findings support the CD data and indicate that β-sheet-rich regions may begin unfolding at lower temperatures compared to intrinsically disordered domains, reinforcing the proteins’ sensitivity to structural perturbations under heat stress. When comparing melting profiles between secondary structure-based and SYPRO Orange-based thermal stability measurements, all three isoforms showed comparable trends in temperature sensitivity (Fig. 2B). These profiles reflect the dynamic, polydisperse nature of sHsps, which are known to regulate liquid–liquid phase separation and respond to heat by partially unfolding or reorganizing to expose hydrophobic patches [33]. Further, the effect of quercetin on tertiary structure stability was evaluated using DSF. Quercetin induced a modest thermal stabilization in PfHsp20a (ΔTm =  + 2 °C) which may be as a result of reduced conformational flexibility due to structural reorganization upon ligand binding. On the other hand, a slight reduction in PfHsp20c (ΔTm = − 1 °C) was observed. However, PfHsp20b was significantly destabilized, with a large thermal shift of − 10 °C (Fig. 2, Table 1). These results indicate that quercetin exerts isoform-specific effects on protein stability, with PfHsp20b being the most structurally vulnerable to the compound.

### Assessing the Ability of *P. falciparum* Hsp20s to Self-associate Using DLS

**Fig. 3:**
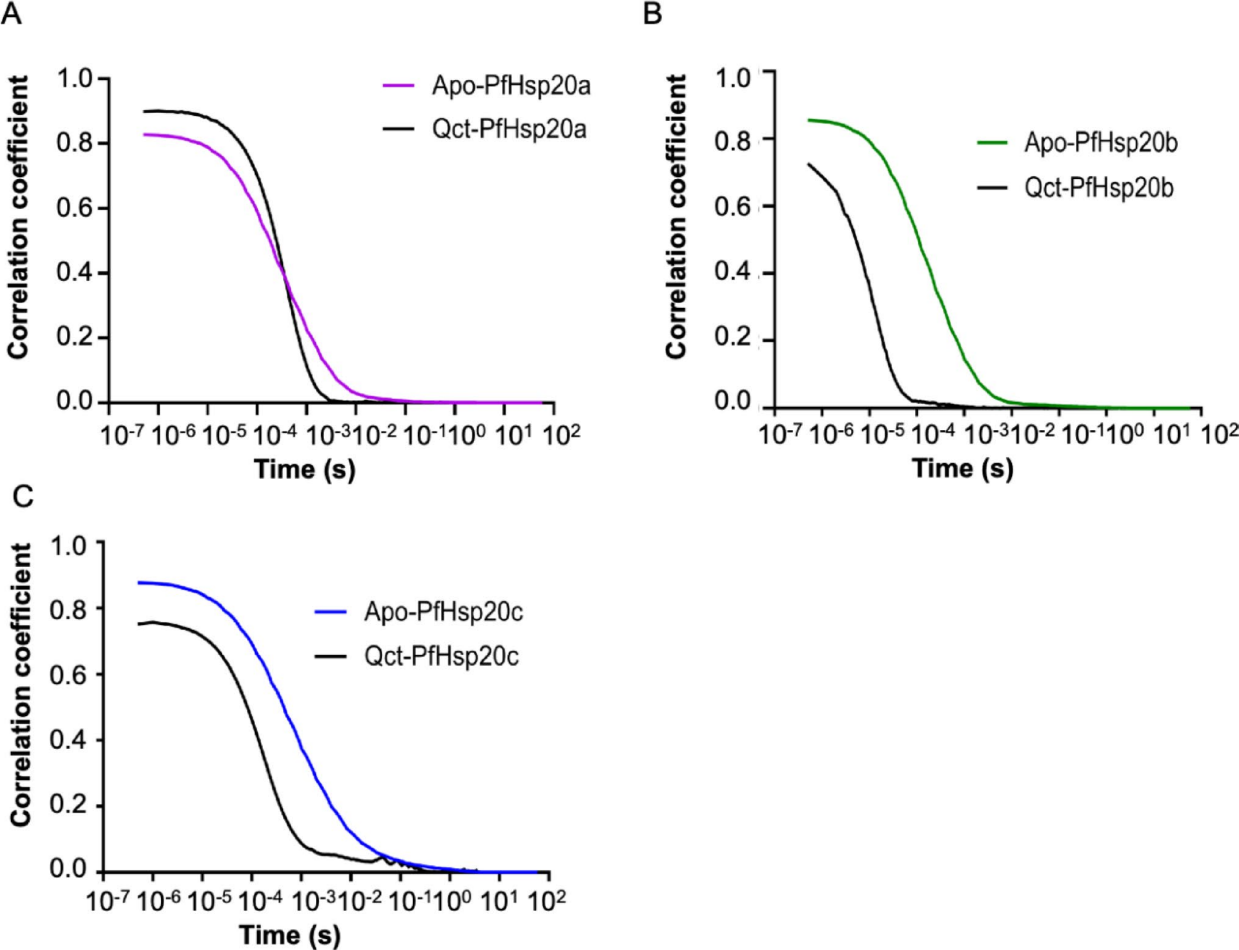
DLS correlation decay curves for *P. falciparum* Hsp20s. The plots represent time-resolved correlation coefficients for the Apo-proteins and quercetin in complex with PfHsp20a (**A**), PfHsp20b (**B**), and PfHsp20c (**C**). The Apo proteins exhibited slower correlation decay compared to their quercetin-bound forms, indicating differences in translational diffusion behaviour. These observations may reflect conformational or hydrodynamic changes upon ligand binding. Time is plotted on a logarithmic scale

The investigation of the size distribution of *P*. *falciparum* Hsp20s in solution was conducted using dynamic light scattering. The DLS data for the *P. falciparum* Hsp20 proteins revealed distinct particle sizes and diffusion rate behaviour, as observed in the different Rh and correlation coefficient analyses (Fig. 3 & Supplementary Figure S4, Table S1). The analysis of the correlation coefficient showed distinct decay rate constants with PfHsp20b (Fig. 3B), having the fastest diffusion rate through the observation volume, followed by PfHsp20a (Fig. 3A) and the slowest being PfHsp20c (Fig. 3C). The relative resultant Rh for PfHsp20b was the smallest at ∼112.3 ± 20.1 nm which is approximately equivalent to a 38-mer oligomer complex (Supplementary Figure S4, Table S1). In contrast, PfHsp20a and PfHsp20c had the largest Rh of ∼227.4 ± 15.5 nm and ∼212.1 ± 30.5 nm, respectively, suggesting these two proteins form larger oligomers in solution (Supplementary Figure S4, Table S1). This is consistent with the slower decay of their correlation function, indicating reduced rate of diffusion across the observation volume. Upon quercetin binding, the decay curves for all three *P. falciparum* Hsp20 proteins exhibited a leftward shift, indicating an increase in diffusion rates due to a reduction in particle size (Fig. 3). The estimated hydrodynamic radius of 138.6 ± 9.5 nm for PfHsp20a, 107.9 ± 16.1 for PfHsp20b and 117 ± 12.8 for PfHsp20c correspond to 40-mer, 37-mer and 35-mer oligomers, respectively (Supplementary Table S1). This suggests that quercetin disrupts larger oligomeric assemblies, likely promoting the formation of smaller subunits. As a control, the hydrodynamic radius (Rh) of BSA was measured at 4.31 ± 2.25 nm (Supplementary Figure S4), aligning with previously reported values in Rh range of 3.2–4.3 nm [35].

### Investigation of the Chaperone Activity of *P. falciparum* Hsp20s

**Fig. 4:**
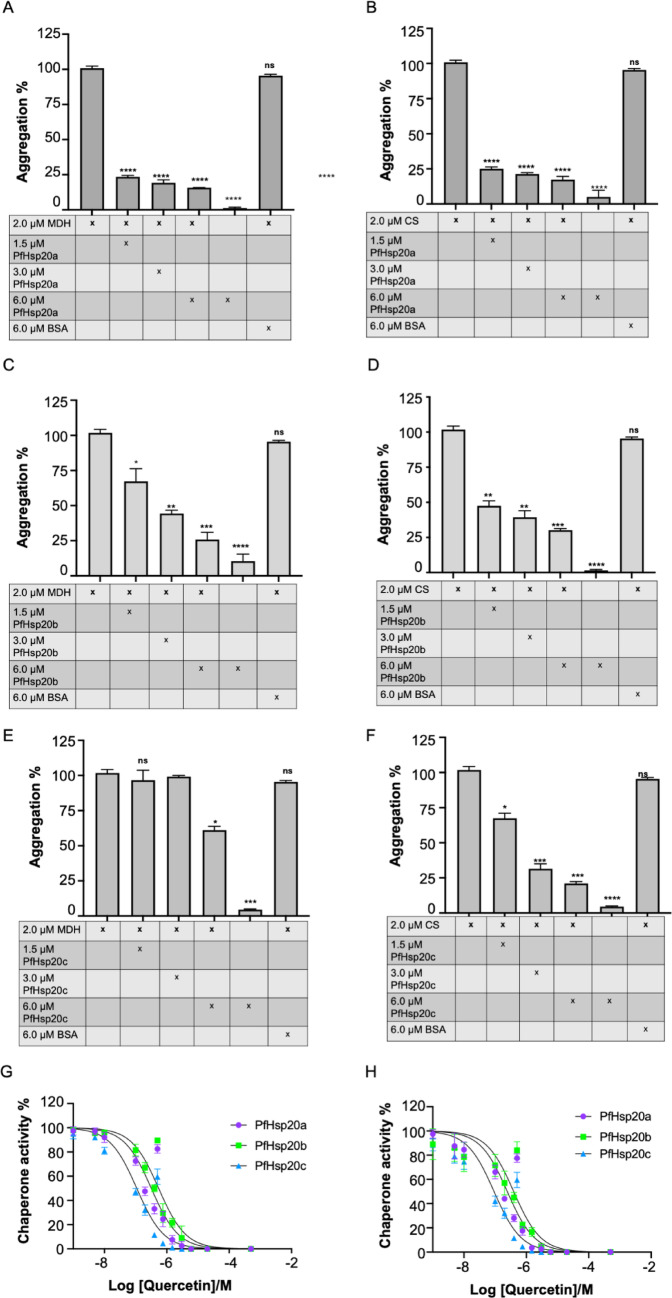
*P. falciparum* Hsp20s supresses thermal induced aggregation of malate dehydrogenase and citrate synthase. The heat induced aggregation of MDH and CS were assessed in vitro at 45 °C. The aggregation suppression capability for PfHsp20a on MDH (**A**) and CS (**B**), PfHsp20b on MDH (**C**) and CS (**D**) and PfHsp20c on MDH (**E**) and CS (**F**). The dose dependent chaperone activity inhibition of the chaperones on MDH (**G**) and CS (**H**). Statistical analysis on three independent repeats was performed using one-way ANOVA, error bars representing standard error are shown, (*) indicating statistically significant differences of the experimental conditions compared to the control (MDH/CS), and ns indicating no significant difference. (*p* ≤ 0.1: *, *p* ≤ 0.01: **, *p* ≤ 0.001: ***, *p* ≤ 0.0001: ****)

The chaperone activity of *P. falciparum* Hsp20 proteins (PfHsp20a, PfHsp20b, PfHsp20c) was assessed using heat-induced aggregation suppression assays with malate dehydrogenase (MDH) and citrate synthase (CS) as model substrates. At 45 °C, both substrates aggregated spontaneously, confirming heat sensitivity and establishing the 100% aggregation baseline. In contrast, the recombinant *P. falciparum* Hsp20 proteins did not self-aggregate at this temperature, in agreement with their thermal stability profiles from CD and DSF assays. BSA, used as a negative control, did not suppress aggregation, while all three PfHsp20s significantly reduced aggregation when added to the reaction (Fig. 4). PfHsp20a showed moderate, concentration-dependent suppression of aggregation, most effective up to 3 × molar excess, (Fig. 4A, B). A similar pattern was seen with PfHsp20b, which also displayed optimal suppression at 3 × concentration for both substrates (Fig. 4C, D). PfHsp20c exhibited weaker, yet consistent, suppression in a concentration-dependent manner, indicating lower overall chaperone capacity (Fig. 4E, F). All isoforms were more effective at suppressing CS aggregation than MDH, suggesting possible substrate preference. When quercetin was introduced, all PfHsp20s showed a concentration-dependent reduction in chaperone activity (Fig. 4G, H). PfHsp20b was the least sensitive (IC_50_ = 0.55 ± 0.10 µM), followed by PfHsp20a (0.32 ± 0.11 µM), while PfHsp20c was the most susceptible with an IC_50_ of 0.11 ± 0.07 µM (Supplementary Table S2). The same trend was observed using CS as substrate. These results suggest that PfHsp20a and PfHsp20b are more effective chaperones at moderate concentrations, while PfHsp20c is intrinsically weaker and more sensitive to quercetin. Overall, despite low sequence identities and varied structural stabilities among these proteins, quercetin is a potent inhibitor of the chaperone function of all three ACD-containing *P. falciparum* Hsp20s.

### Molecular Docking Analysis

**Fig. 5:**
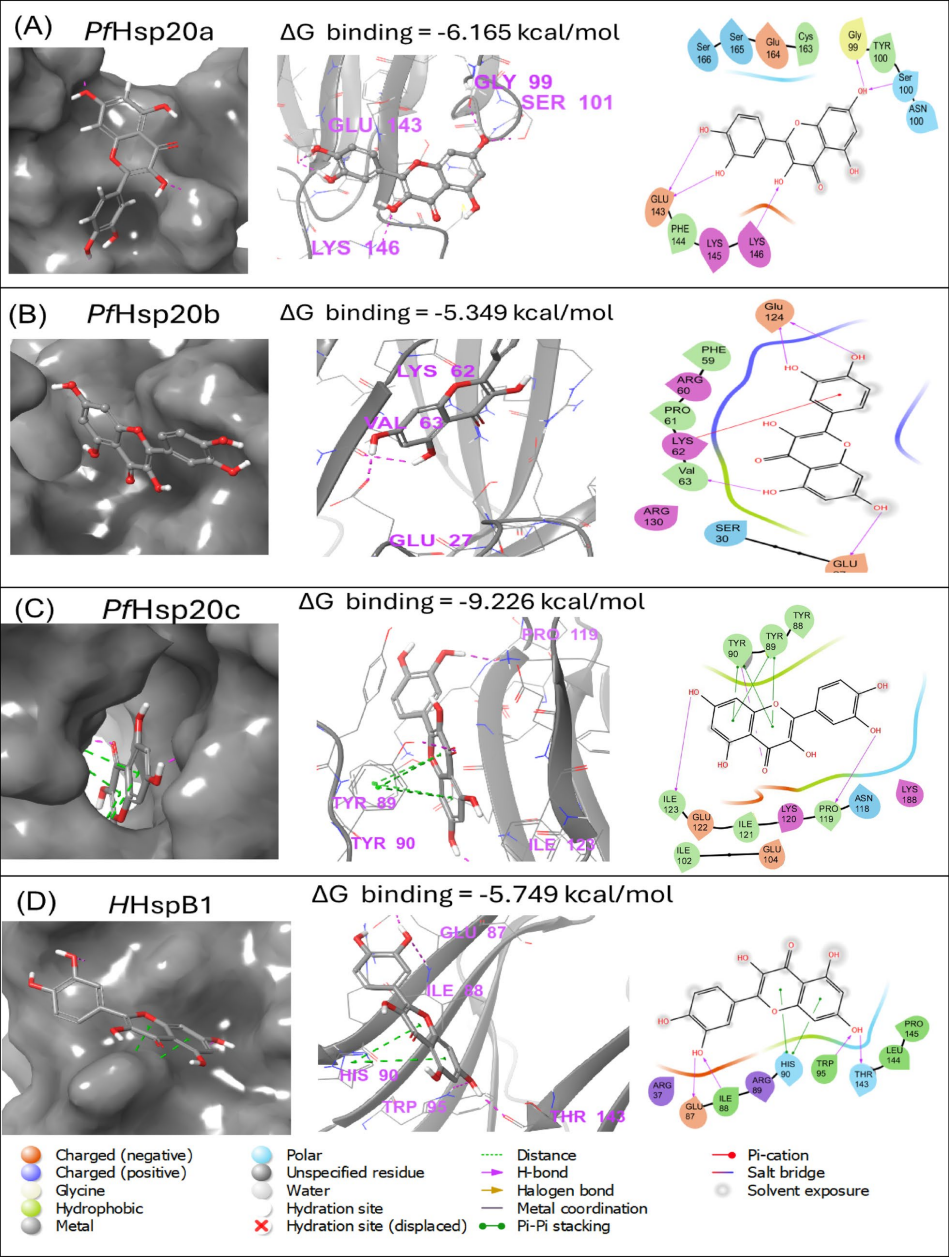
Binding poses of quercetin in *P. falciparum* Hsp20s. Interactions of quercetin with PfHsp20a (**A**), PfHsp20b (**B**), PfHsp20c (**C**) and human HspB1 (**D**) are shown. The ligand–receptor interaction maps are represented showing bonds and interaction/contacts. Hydrogen bonds (blue), Pi–Pi stacking (green) and hydrophobic contacts (grey)

Molecular docking was conducted to further investigate the potential interacting residues between the quercetin and the Hsp20 proteins using the Schrödinger Maestro software. The docking poses and free binding energy values showed that quercetin bound with moderate affinity to the *P. falciparum* Hsp20s (Fig. 5). The association of PfHsp20a was with a binding energy of − 6.165 kcal/mol (Fig. 5A). Similarly, PfHsp20b had a binding energy of − 5.349 kcal/mol (Fig. 5B) and the highest binding energy were with PfHsp20c at − 9.226 kcal/mol (Fig. 5C). As a control we also noted that human HspB1 bound quercetin bound at − 5.749 kcal/mol (Fig. 5D). The three protein PfHsp20a, PfHsp20b and PfHsp20c, all interacted with quercetin through unique residues for each protein (Supplementary Table S3). In the analysis of the binding sites, we identified regions on the NTD and ACD as being crucial for substrate binding in small Hsps which are in line with previous findings on the human homologues [6].

### Molecular Dynamics Simulations

**Fig. 6:**
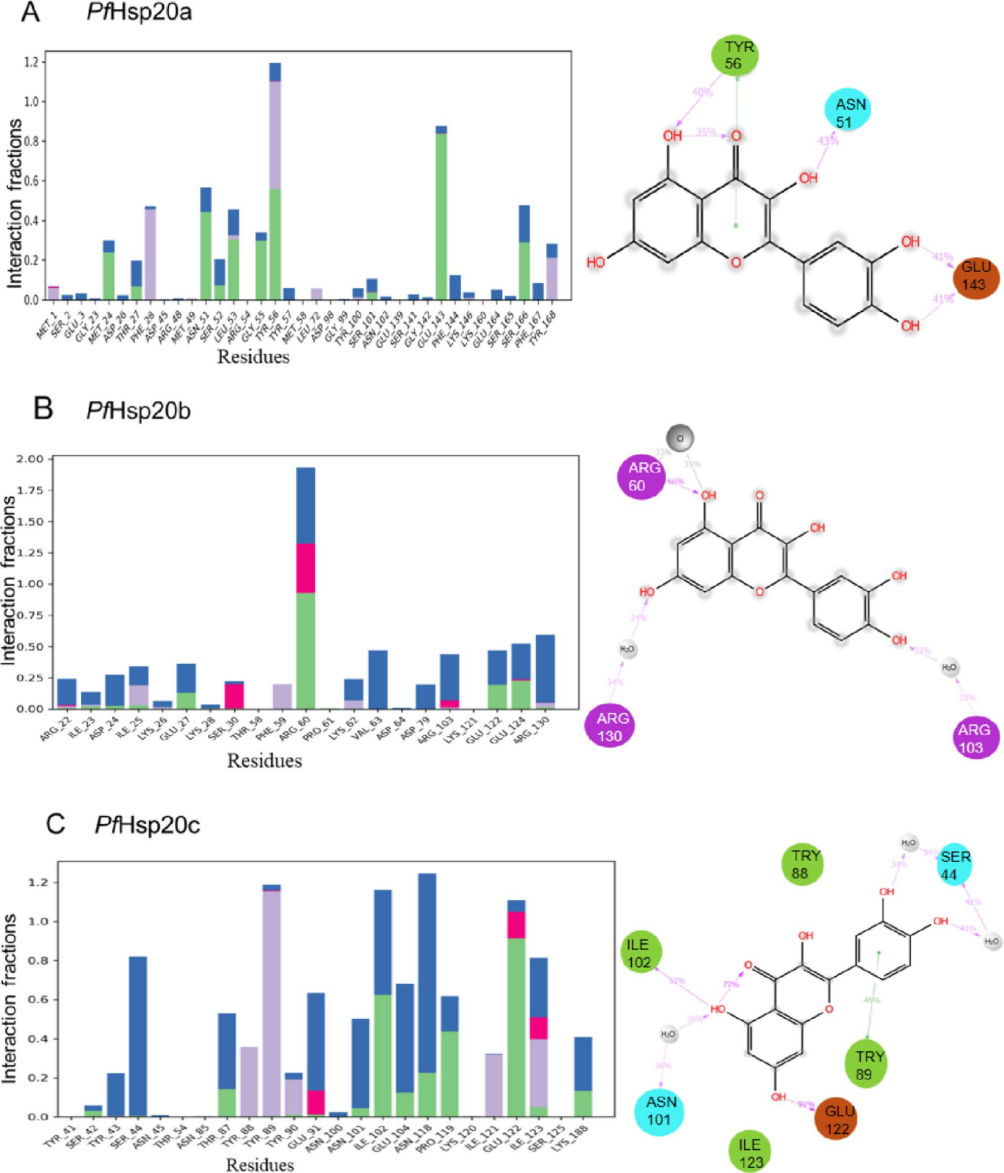
Analysis of residues implicated in *P. falciparum* Hsp20-quercetin complex stability during the MD simulations. Analysis of the type of interactions between quercetin with **A** PfHsp20a, **B** PfHsp20b and **C** PfHsp20c, the insert highlights the ligand atom interactions with the protein residues that occurred more than 30% of the simulation period within the active site. The right panel show the 2-D rendering of the interacting residues between the protein and ligand

Molecular dynamics (MD) simulations using the Desmond algorithm were conducted to assess the stability and conformational dynamics of *P. falciparum* Hsp20 isoforms (PfHsp20a, PfHsp20b, PfHsp20c) in both apo- and quercetin-bound forms (Supplementary Table S4). The simulations revealed that quercetin binding affected protein compactness to varying degrees, with PfHsp20b showing the greatest increase in compactness (Rg change of 1.83 Å), followed by PfHsp20a (1.32 Å), and minimal impact on PfHsp20c (0.23 Å) (Table 2). Root mean square deviation (RMSD) analysis indicated that PfHsp20b and PfHsp20c became more structurally stable upon quercetin binding, with reduced RMSD values of − 6 Å and − 0.57 Å respectively, while PfHsp20a exhibited a rise in RMSD by 2.9 Å, suggesting lower stability (Table 2). Ligand RMSD values supported these trends: PfHsp20b showed the most stable binding (5.44 ± 1.67 Å), followed by PfHsp20c (6.34 ± 0.91 Å), while PfHsp20a displayed the least stable interaction (8.74 ± 1.78 Å) with large fluctuations in the initial 40 ns (Table 2). Root mean square fluctuation (RMSF) analysis showed that quercetin induced the greatest flexibility in PfHsp20a (12.87 Å), followed by PfHsp20c (6.80 Å), and least in PfHsp20b (4.52 Å), indicating the latter’s enhanced stability in the bound state (Table 2). Interaction profiling revealed that PfHsp20a stability was driven by hydrogen bonds involving Asn51, Tyr56, and Glu143, and π–π stacking with Tyr56 (Fig. 6A). PfHsp20b interactions were stabilized by Arg60 hydrogen bonding and water bridges with Arg103 and Arg130 (Fig. 6B), while PfHsp20c stability was mediated through interactions involving Ser44, Asn101, Ile102, Glu122, and π–π stacking via Tyr89 (Fig. 6C). Overall, these findings demonstrate that quercetin forms the most stable complex with PfHsp20b, moderately stabilizes PfHsp20c, and binds least effectively to PfHsp20a. This stability hierarchy aligns with experimental results from DSF and DLS assays, suggesting that quercetin may disrupt Hsp20 function and structure, supporting its potential as an antimalarial lead compound.

**Table 2:** The MD simulation binding parameters

Receptor	Ligand	Rg mean ± SD/Å	RMSD of Cα ± SD/Å	Ligand RMSD ± SD/Å	RMSF mean ± SD/Å
PfHsp20a	–	24.82 ± 1.82	15.39 ± 1.83	–	4.55 ± 2.77
	Quercetin	23.50 ± 4.19	17.79 ± 4.68	8.74 ± 1.78	17.37 ± 5.73
PfHsp20b	–	20.81 ± 1.09	18.54 ± 3.32	–	6.98 ± 2.66
	Quercetin	18.98 ± 0.61	12.49 ± 1.23	5.44 ± 1.67	11.15 ± 3.70
PfHsp20c	–	20.70 ± 0.84	12.66 ± 3.23	–	3.67 ± 2.06
	Quercetin	20.47 ± 0.84	12.09 ± 1.20	6.34 ± 0.91	10.50 ± 3.90

The radius of gyration (Rg), root mean square deviation (RMSD) of Cα atoms, ligand RMSD and the root mean square fluctuation (RMSF) values between the apo and ligand binding are shown over 100 ns. The graphical representation of the traces over the simulation time are shown in Supplementary Figure S5

### Assessing the Liquid–Liquid Phase Separation Propensity of *P. falciparum* Hsp20s

**Fig. 7:**
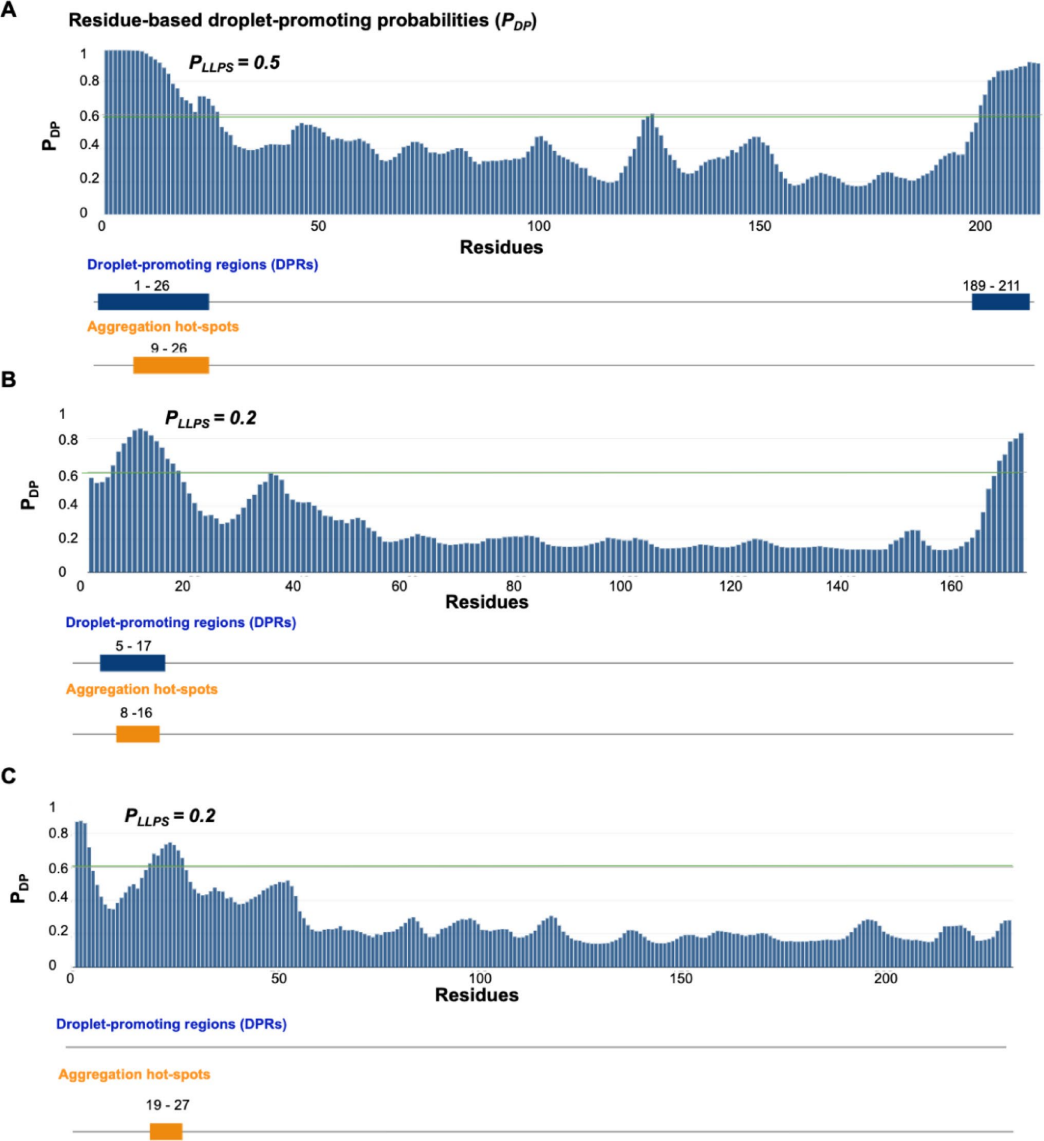
The droplet promoting likelihood profiles for *P. falciparum* Hsp20s. The residue-based droplet promoting probabilities (pDP) values were plotted against the amino acid sequence for PfHsp20a (**A**), PfHsp20b (**B**) and PfHsp20c (**C**). The regions which with a high propensity to promote droplet formation and aggregation hot spots are highlighted indicating the residue numbers. Droplet promoting probability (*P*_*LLPS*_) of native *P. falciparum* Hsp20s values are shown, respectively

Using the FuzDrop tool, the potential for *P. falciparum* Hsp20s proteins to undergo liquid–liquid phase separation (LLPS) was assessed, as sHsps from other organisms are known to exhibit this behaviour [32, 33]. All three parasite sHsps had PLLPS scores below the 0.6 threshold [36], indicating no spontaneous droplet formation under native conditions (Fig. 7). Among them, PfHsp20a showed the highest LLPS propensity with a score of 0.5, suggesting it may act as a droplet client in the presence of cofactors or under altered conditions such as divalent ion presence (Fig. 7A). PfHsp20b and PfHsp20c both scored 0.2 (Fig. 7B, C). The unstructured N-terminal (NTD) and C-terminal (CTD) domains were identified as key regions for droplet promotion and aggregation. PfHsp20a showed overlapping droplet-promoting and aggregation hotspots in residues 9–26 (NTD) and 189–211 (CTD). PfHsp20b’s hotspots were confined to the NTD, while PfHsp20c lacked droplet-promoting regions. These findings suggest low LLPS propensity for parasite sHsps under native conditions.

### *P. falciparum* Growth Inhibition Analysis of Quercetin

After confirming that quercetin is a potent inhibitor of the parasite Hsp20s, we then sought to determine its inhibitory activity to parasite growth. *P. falciparum* NF54 and Dd2 strains were grown in the presence of quercetin. As controls we observed that chloroquine and artemisinin inhibited parasite growth at the expected levels [37]. We determined that quercetin exhibited moderate parasite growth inhibition with IC_50_ of 5.43 ± 1.45 µM and 7.16 ± 1.03 µM against NF54 and Dd2 parasite strains (Table 3). We also noted that quercetin had a resistance index of 1.32 which was lower than chloroquine but higher than artemisinin. This suggest that the quercetin targeted the same mechanism of action in both drug sensitive and resistant strains. This partly explain the moderate activity of quercetin could be through potentially abrogating parasite growth through its inhibition on the ACD containing Hsp20s.

**Table 3:** *P. falciparum* growth inhibition analysis

Compound	IC_50_ ± SD µM		RI
	*P. falciparum* strain		
	Nf54	Dd2	
Quercetin	5.43 ± 1.46	7.16 ± 1.03	1.32
Chloroquine	0.009 ± 0.002	0.194 ± 0.011	21.67
Artesunate	0.004 ± 0.002	0.005 ± 0.001	1.00

The RI is the resistance index between the two drugs. Data shown are the mean of three independent experiments conducted in duplicate (n = 2) and standard deviation is shown

### Pharmacokinetics/Pharmacodynamics of Quercetin

In silico ADMET analysis revealed that quercetin possesses moderate aqueous solubility (log S = − 3.152) and Caco-2 cell permeability (log Papp = 1.021), along with high predicted intestinal absorption (75.34%) but limited skin permeability (log Kp = − 2.735) (Supplementary Table S5). It is a substrate of the P-glycoprotein (P-gp) efflux transporter, suggesting potential modulation of its absorption and bioavailability. Distribution parameters indicated low volume of distribution (log Vd = 0.223) and limited plasma protein binding (unbound fraction = 0.061). The compound exhibited poor brain penetration (log BB = − 1.377; log PS = − 3.475). Metabolic predictions showed quercetin inhibits CYP1A2 and CYP2C19 but is not a substrate of CYP2D6, nor an inhibitor of CYP2C9, CYP2D6, or CYP3A4, indicating a selective potential for drug–enzyme interactions. It demonstrated moderate clearance (log CL = 0.663 mL/min/kg) and was not a substrate for renal OCT2 transporters, implying passive excretion. Toxicity assessments were favourable: quercetin tested negative in the Ames mutagenicity model and showed no predicted hepatotoxicity, skin sensitization, or hERG channel inhibition. Quercetin complied with Lipinski’s Rule of Five (MW = 302.24 g/mol; 5 H-bond donors; 7 H-bond acceptors; TPSA = 131.36 Å^2^), with no violations and a predicted oral bioavailability score of 0.55, supporting its drug-likeness profile.

## Discussion

*P. falciparum* expresses three Hsp20s, PfHsp20a, PfHsp20b, and PfHsp20c, each containing the conserved ACD [19]. While their specific roles remain unclear, analogous proteins in *P. berghei* are essential for sporozoite motility and hepatocyte invasion [38, 39]. In other organisms, sHsps serve in proteostasis and interact with larger chaperones like Hsp70 and Hsp90 [6, 40], suggesting that *P. falciparum* Hsp20s may also be critical for stress tolerance and survival across the parasite’s complex lifecycle. These functions make them attractive antimalarial drug targets.

Despite the known expression challenges of malarial proteins [41], all three *P. falciparum* Hsp20s were successfully expressed and purified without co-chaperone assistance (Supplementary Figure S1). Structural analyses revealed diversity among isoforms: while all shared a β-sheet-rich ACD, PfHsp20b appeared more disordered in its N- and C-terminal domains (Fig. 1), similar to intrinsically disordered proteins like Hsp22 [42]. Thermal assays confirmed all three isoforms are heat-resistant, with PfHsp20a showing the highest stability and PfHsp20b the least (Fig. 1D, E, F). Although DSF produced different melting points compared to CD, both methods indicated quercetin reduced thermal stability across all three isoforms, most significantly in PfHsp20b (Figs. 1G, 2, Table 1) [43].

Oligomerization is key to sHsp function [4, 10–12], and our DLS studies revealed that PfHsp20b forms smaller oligomers than PfHsp20a or PfHsp20c (Fig. 3, Supplementary Table S1). Notably, quercetin altered oligomer size distribution, especially reducing PfHsp20b oligomer size. While smaller oligomers are often associated with enhanced activity [44], in this study, quercetin-induced size reduction corresponded with decreased chaperone activity, indicating structural disruption may impair function, consistent with findings in other systems [45].

Functionally, all three *P. falciparum* Hsp20s suppressed heat-induced aggregation of MDH and CS, though their effectiveness varied. PfHsp20a was most efficient at lower concentrations, followed by PfHsp20b, while PfHsp20c had the least chaperone activity (Fig. 4A–F). This suggests that PfHsp20a likely forms more active oligomeric states under physiological conditions. Additionally, none of the parasite Hsp20s exhibited high propensity for spontaneous liquid–liquid phase separation under native conditions (Fig. 7), though PfHsp20a had the highest potential among them. This suggest a different mechanism of substrate aggregation sequestration that needs to be further explored. Quercetin inhibited the chaperone function of all three proteins in a dose-dependent manner (Fig. 4G, H, Supplementary Table S2), with PfHsp20c being most sensitive followed by PfHsp20a, and PfHsp20b being least affected. These functional disparities reinforce the hypothesis that each *P. falciparum* Hsp20 isoform has evolved for distinct roles within the parasite.

The molecular docking and MD simulations provided deeper insight into quercetin binding. While docking scores suggested strongest affinity for PfHsp20c, MD simulations indicated that the PfHsp20b-quercetin complex was the most stable over time, followed by PfHsp20c, with PfHsp20a showing the highest structural fluctuation (Fig. 6). Radius of gyration analysis confirmed that quercetin increased compactness of PfHsp20b but had minimal effect on PfHsp20a and PfHsp20c (Table 2, Supplementary Figure S5). RMSD and RMSF analyses showed reduced flexibility in PfHsp20b and PfHsp20c upon quercetin binding, whereas PfHsp20a remained structurally dynamic. Residue-level interaction analysis revealed that quercetin formed stable hydrogen bonds, hydrophobic interactions, and water bridges, predominantly within the NTD and ACD regions (Fig. 6). In PfHsp20a, the key interaction was a π–π stacking with Tyr56. For PfHsp20b, critical interactions included hydrogen bonding with Arg60 and water bridging with Arg103 and Arg130. In PfHsp20c, water bridges involving Ser44 and Asn101, as well as hydrogen bonds with Ile102 and Glu122, stabilized the complex.

In vitro antiplasmodial assays demonstrated that quercetin moderately inhibited both chloroquine-sensitive and -resistant *P. falciparum* strains, with IC_50_ values ranging between 5 and 10 µM (Table 3). Although less potent than conventional antimalarials like chloroquine or artesunate, quercetin’s favourable ADMET profile and selective inhibition of *P. falciparum* function [46] underscore its potential as a lead compound for further optimization. It is plausible that the interaction of quercetin with the parasite Hsp20s resulting in their functional abrogation, contributed in part to its antiplasmodial effects. However, our findings suggest a potential interaction between quercetin and the *P. falciparum* Hsp20s, it remains unclear to what extent quercetin’s antiplasmodial activity is directly mediated by interaction with *P. falciparum* Hsp20 proteins. Considering the compound’s pleiotropic nature, future studies involving functional validation, such as genetic knockdowns or phenotypic correlation assays, will be important to establish a direct causal relationship.

Our study provides the first comprehensive characterization of *P. falciparum* Hsp20 proteins, revealing their structural integrity, thermotolerance, chaperone function, and susceptibility to quercetin inhibition. The findings demonstrate that these sHsps, particularly PfHsp20a and PfHsp20b, are essential for parasite proteostasis and represent viable molecular targets for antimalarial therapy. Considering that these sHsps are nucleotide independent chaperones which may act as a first line of defence against protein misfolding under ATP depleted stressful conditions. Future research should be prioritised to focus on understanding their precise roles in actin regulation, stress response, and interaction with other chaperones. High-resolution structural studies and small molecule screening could pave the way for developing novel Hsp20-targeted interventions against malaria.

### Limitations to the Study

The use of full-length protein in our current study does not reflect the final biologically functioning protein in the parasite as some post-translational modifications and cleavages to achieve fully mature protein could not be mimicked in the expression system used in our current study. Therefore, the findings should be interpreted in context with the construct used.

## Electronic Supplementary Material

Below is the link to the electronic supplementary material.

Supplementary Material 1

## Data Availability

Data is provided within the manuscript or supplementary information files.
