# Supplementary material for: Comparative Characterization of Plasmodium falciparum Small Heat Shock Proteins and Their Inhibition by Quercetin (3,3′,4′,5,7-Pentahydroxyflavone)
Source: Protein J. 2025 Jul 18;44(5):580–97. doi: 10.1007/s10930-025-10281-w (PMC12457548; doi:10.1007/s10930-025-10281-w)
Supplement: Supplementary file 1 — Supplementary Material 1 [file 10930_2025_10281_MOESM1_ESM.docx]

Supplementary Data

**Supplementary Table S1: Estimation of the hydrodynamic radius of *P. falciparum* Hsp20 oligomers**

| Protein | DLS (mean ± SD) | 3D structure (nm) | Estimated Oligomer size (DLS/Rh) |
| --- | --- | --- | --- |
| PfHsp20a | 227.4 ± 15.5 | 3.4 | 66.8 ± 4.6 |
| PfHsp20a + quercetin | 138.6 ± 9.5 |  | 40.6 ± 2.6 |
| PfHsp20b | 112.3 ± 20.1 | 2.9 | 38.6 ± 6.9 |
| PfHsp20b + quercetin | 107.9 ± 16.1 |  | 37.2 ± 8.7 |
| PfHsp20c | 212.1 ± 30.5 | 3.3 | 64.2 ± 9.2 |
| PfHsp20c + quercetin | 117 ± 12.8 |  | 35.5 ± 4.4 |

The DLS estimated sizes were converted to oligomer sizes as a ratio of the predicted 3D structure of each protein using the Fluidic sciences webserver and the predicted monomeric AlphaFold structures (Supplementary Figure S2). The standard deviation of the mean from three technical repeats of the DLS analysis is shown.

**Supplementary Table S2. The inhibition of the *P. falciparum* Hsp20s chaperone activity with quercetin**

| Chaperone | Aggregation suppression inhibition IC**₅₀** ±SEM / µM | |
| --- | --- | --- |
|  | MDH | CS |
| PfHsp20a | 0.32 ± 0.11 | 0.24 ± 0.15 |
| PfHsp20b | 0.55 ± 0.10 | 0.39 ± 0.14 |
| PfHsp20c | 0.11 ± 0.07 | 0.09 ± 0.1 |

The standard error of the mean from three biological repeats of the dose response analysis is shown.

**Supplementary Table S3. Docking scores for *P. falciparum in* complex with quercetin**

| **Receptor/ligand name** | **Interacting residues within 4 Å** | **∆G binding energy (kcal/mol)** |
| --- | --- | --- |
| PfHsp20a | Gly 99 Ser 101 Glu 143 Lys 146 | -6.165 |
| PfHsp20b | Glu 27 Lys 62 Val 63 | -5.349 |
| PfHsp20c | Tyr 89 Tyr 90 Pro 119 Ile 123 | -9.226 |
| HspB1 | Glu 87 Ile 88 His 90 Trp 95 Thr 143 | -5.749 |

**Supplementary Table S4. Quality assessment of the simulated systems over a 100ns period**

| **Systems simulated** | **Parameter** | **Average** | **Standard deviation** | **Slopes (ps-1)** |
| --- | --- | --- | --- | --- |
| **QCT-PfHsp20a** | Total energy (Kcal/mol) | -269329.67 | 128.83 | -0.00 |
|  | Potential energy (Kcal/mol) | -327913.22 | 109.38 | -0.00 |
|  | Temperature (K) | 298.72 | 0.44 | 0.00 |
|  | Pressure (bar) | 0.58 | 27.14 | 0.00 |
|  | Volume (Å³) | 985680.80 | 574.03 | 0.00 |
|  |  |  |  |  |
| **QCT-PfHsp20b** | Total energy (Kcal/mol) | -168037.70 | 105.38 | -0.00 |
|  | Potential energy (Kcal/mol) | -204821.07 | 90.12 | -0.00 |
|  | Temperature (K) | 298.71 | 0.50 | 0.00 |
|  | Pressure (bar) | 0.74 | 34.79 | 0.00 |
|  | Volume (Å³) | 616820.90 | 455.60 | -0.00 |
|  |  |  |  |  |
| **QCT-PfHsp20c** | Total energy (Kcal/mol) | -216550.07 | 120.62 | 0.00 |
|  | Potential energy (Kcal/mol) | -264369.67 | 102.07 | 0.00 |
|  | Temperature (K) | 298.72 | 0.47 | 0.00 |
|  | Pressure (bar) | 0.93 | 31.09 | 0.00 |
|  | Volume (Å³) | 801145.13 | 529.46 | 0.00 |

**Supplementary Table S5.** **Predicted ADME/T properties of quercetin**

| **Properties** | **Quercetin** | **Normal Range ^A^** |
| --- | --- | --- |
| **Absorption** | | |
| Water solubility (log mol/L) | -3.152 | -6.5 to 0.5 |
| Caco2 permeability (log Papp in 10⁻⁶ cm/s) | 1.021 | ≥ 0.9 (good permeability) |
| Intestinal absorption (human) (% Absorbed) | 75.335 | Low < 25%, High > 85% |
| Skin permeability (log Kp) | -2.735 | -8.0 to -1 (log Kp) |
| P-glycoprotein substrate | Yes | N/A |
| P-glycoprotein I inhibitor | No | N/A |
| P-glycoprotein II inhibitor | No | N/A |
| **Distribution** | | |
| VDss (human) (log L/kg) | 0.223 | 0.04 - 1.25 (moderate distribution) |
| Fraction unbound (human) (Fu) | 0.061 | 0.01 - 0.5 (acceptable) |
| BBB permeability (log BB) | -1.377 | -3.0 to 1.2 |
| CNS permeability (log PS) | -3.475 | ≤ -2.0 |
| **Metabolism** | | |
| CYP2D6 substrate | No | N/A |
| CYP1A2 inhibitor | Yes | N/A |
| CYP2C19 inhibitor | Yes | N/A |
| CYP2C9 inhibitor | No | N/A |
| CYP2D6 inhibitor | No | N/A |
| CYP3A4 inhibitor | No | N/A |
| **Excretion** | | |
| Total Clearance (log ml/min/kg) | 0.663 | 0.5 - 1.2 (moderate clearance) |
| Renal OCT2 substrate | No | N/A |
| **Toxicity** | | |
| AMES toxicity | No | N/A |
| Max. tolerated dose (human) (log mg/kg/day) | 1.159 | > 0.5 (acceptable) |
| hERG I inhibitor | No | N/A |
| hERG II inhibitor | No | N/A |
| Hepatotoxicity | No | N/A |
| Skin Sensitisation | No | N/A |
| **Drug-likeness properties** | | |
| Molecular weight (g/mol) | 302.24 | ≤ 500 g/mol |
| Number of H-bond acceptors | 7 | < 10 |
| Number of H-bond donors | 5 | < 5 |
| Number of rotatable bonds | 1 | ≤ 8 |
| Topological polar surface area (TPSA) (Å²) | 131.36 | < 140 Å² |
| Log Po/w (iLogP) | 1.63 | < 5 |
| Lipinski's rule of five violation | 0 Violation | ≤ 1 violation |
| Bioavailability score | 0.55 | ≥ 0.55 |

Papp: apparent permeability coefficient, AMES: assay of the ability of a chemical compound to induce mutations in DNA, Kp: skin permeability, VDss: distribution volume, Fu: fraction unbound, BBB: blood–brain barrier, BB: blood–brain, CNS: central nervous system, PS: permeability-surface area, OCT2: Organic cation transporter 2, hERG: human ether-a-go-go-related gene. NA: Not applicable. Ref: 46.

**Supplementary Figure S1:** **Expression and purification of recombinant proteins.** The SDS-PAGE analysis of the expression and purification of **(A)** PfHsp20a, **(B)** PfHsp20b and **(C)** PfHsp20c. The western blot analysis using His probe (lower panels). The lane M represent molecular weight marker (kDa), lane C - total cell lysate of *E coli* XL1 blue cells transformed with pQE30 vector only. Lane 0-6 the hourly samples of the total cell lysates of *E. coli* cells transformed with respective plasmid, lane E - the elution fraction of the purified recombinant proteins.

**Supplementary Figure S2. The comparative structure analysis of PfHsp20s**. The superimposed 3D structure of A) PfHsp20a with PfHsp20b, B) PfHsp20a with PfHsp20c showing the conservation ACD domain and varying NTD and CTD domains. C) The surface orientation or the structures rendered at 90 degree rotation. D) The multiple sequence alignment of the parasite Hsps20 showing the conserved residues identical in white text on red background and those similar in red text on white background and the non-conserved residues are in black text on white background. The respective sequence identity percentages are indicated at the end of the sequences.

**Supplementary Figure S3. The CD spectra of *P. falciparum* Hsp20**s. The temperature response on the CD spectra for PfHsp20a (A), PfHsp20b (B) and PfHsp20c (C). The temperature of the suspended protein was increased from 25 °C to 90 °C whilst monitoring the changes in ellipticity at 190 – 260 nm.

**Supplementary Figure S4. Dynamic Light Scattering analysis of PfHsp20 isoforms and their interaction with Quercetin . The** DLS profiles show the hydrodynamic size distributions of the recombinant PfHsp20a (A), PfHsp20b (B), and PfHsp20c (C), in the absence and presence of Quercetin (QCT, red). Bovine Serum Albumin (BSA, black), with a known hydrodynamic radius served as control. PfHsp20a (purple), PfHsp20b (green), and PfHsp20c (blue) each display large oligomeric assemblies under native conditions. Upon addition of Quercetin (red), all three proteins exhibit a shift toward smaller hydrodynamic radii, indicating ligand-induced structural reorganization or oligomer dissociation.

**Supplementary Figure S5. Analysis of protein quercetin-Hsp20 complex stability during the MD simulations**. **(A)** The Rg of Cα atoms **(B)** The RMSD plot of apo-Hsp20s and quercetin bound complexes over a 100ns simulation period **(C)** The Ligand RMSD graph showing the stability of the compounds with respect to the proteins binding sites. Images were generated from the simulation interaction diagram algorithm in Maestro v13.1.
